# Morphological and Molecular Characterizations of Psychrophilic Fungus *Geomyces destructans* from New York Bats with White Nose Syndrome (WNS)

**DOI:** 10.1371/journal.pone.0010783

**Published:** 2010-05-24

**Authors:** Vishnu Chaturvedi, Deborah J. Springer, Melissa J. Behr, Rama Ramani, Xiaojiang Li, Marcia K. Peck, Ping Ren, Dianna J. Bopp, Britta Wood, William A. Samsonoff, Calvin M. Butchkoski, Alan C. Hicks, Ward B. Stone, Robert J. Rudd, Sudha Chaturvedi

**Affiliations:** 1 Mycology Laboratory, Wadsworth Center, New York State Department of Health, Albany, New York, United States of America; 2 Department of Biomedical Sciences, School of Public Health, University at Albany, Albany, New York, United States of America; 3 Anatomic Pathology Core, Wadsworth Center, New York State Department of Health, Albany, New York, United States of America; 4 Bacteriology Laboratory, Wadsworth Center, New York State Department of Health, Albany, New York, United States of America; 5 Rabies Laboratory, Wadsworth Center, New York State Department of Health, Albany, New York, United States of America; 6 Electron Microscopy Core, Wadsworth Center, New York State Department of Health, Albany, New York, United States of America; 7 Pennsylvania Game Commission, Harrisburg, Pennsylvania, United States of America; 8 Bureau of Wildlife, New York State Department of Environmental Conservation, Albany, New York, United States of America; 9 Wildlife Pathology Unit, New York State Department of Environmental Conservation, Albany, New York, United States of America; Massachusetts General Hospital, United States of America

## Abstract

**Background:**

Massive die-offs of little brown bats (*Myotis lucifugus*) have been occurring since 2006 in hibernation sites around Albany, New York, and this problem has spread to other States in the Northeastern United States. White cottony fungal growth is seen on the snouts of affected animals, a prominent sign of White Nose Syndrome (WNS). A previous report described the involvement of the fungus *Geomyces destructans* in WNS, but an identical fungus was recently isolated in France from a bat that was evidently healthy. The fungus has been recovered sparsely despite plentiful availability of afflicted animals.

**Methodology/Principal Findings:**

We have investigated 100 bat and environmental samples from eight affected sites in 2008. Our findings provide strong evidence for an etiologic role of *G. destructans* in bat WNS. (i) Direct smears from bat snouts, Periodic Acid Schiff-stained tissue sections from infected tissues, and scanning electron micrographs of bat tissues all showed fungal structures similar to those of *G. destructans.* (ii) *G. destructans* DNA was directly amplified from infected bat tissues, (iii) Isolations of *G. destructans* in cultures from infected bat tissues showed 100% DNA match with the fungus present in positive tissue samples. (iv) RAPD patterns for all *G. destructans* cultures isolated from two sites were indistinguishable. (v) The fungal isolates showed psychrophilic growth. (vi) We identified *in vitro* proteolytic activities suggestive of known fungal pathogenic traits in *G. destructans*.

**Conclusions/Significance:**

Further studies are needed to understand whether *G. destructans* WNS is a symptom or a trigger for bat mass mortality. The availability of well-characterized *G. destructans* strains should promote an understanding of bat–fungus relationships, and should aid in the screening of biological and chemical control agents.

## Introduction

Bats, which are ‘keystone species’ in many ecosystems, play notable roles in plant pollination, forest regeneration and control of insect populations [1], [2]. Bats are important to human health as they are reservoirs or carriers for rabies and other viruses, parasites, and pathogenic fungi [3], [4], [5], [6], [7]. Hibernation is believed to be an important adaptation in bats that may contribute to their exceptional longevity [8]. The common little brown bat (*Myotis lucifugus*) hibernates, along with the endangered Indiana bat (*Myotis sodalis*), in many hibernacula in the Northeastern United States, including caves and mines in upstate New York [9], [10]. Hibernating bats can suffer significant mortality due to adverse environmental conditions such as freezing or flooding, as well as human activities including visitation and pesticide applications [11], [12]. No mass mortality was reported until recently from bat sites that had been surveyed for almost three decades by the New York State Department of Environmental Conservation. Recently, however little brown bats have been found to be dying in large numbers at many hibernation sites in upstate New York [13]. This problem has spread to other States in the Northeastern US (Figure S1).

The first noticeable bat declines in winter hibernacula were observed in 2006 from Hailes and Knox Caves, and Gage and Schoharie Caverns, situated within a 12-km radius of the Albany metropolitan area in upstate New York. In 2007, the Rabies Laboratory at the Wadsworth Center of the New York State Department of Health also received unusually large number of bat submissions for rabies testing. The large numbers of bat deaths were considered alarming, because no exceptional events, whether environmental or anthropogenic, were reported from the affected areas during this period. Most diseased animals displayed what came to be known as ‘white nose syndrome’ (WNS), which includes a prominent sign of white cottony growth around the snout. These animals were examined by a number of laboratories for the recovery of pathogenic microbes and/or the presence of toxic chemicals, and a number of animals were selected for necropsy to establish the cause of death. An important breakthrough in these investigations was reported by Blehert et al. [14], who cultured a fungus similar to *Geomyces* species from tissues of bats afflicted with WNS. Subsequently, this fungus was named as a new species, *Geomyces destructans* [15], although it is closely related to other psychrophilic (cold-loving) species of *Geomyces* [16]. A recent publication described the recovery of the fungus and related findings from bats in New York and Connecticut [17]. Very recently, Puechmaille et al. [18] described the isolation of *G. destructans* from an apparently healthy bat (*M. myotis*) in France. A common theme of these mycological investigations is the relatively sparse isolations of the fungus from bats despite the ready availability of bats with WNS. Also, no details were provided in the aforementioned publications as to why recovery rates are so low, or what specific requirements (if any) are needed, other than incubation at 4°-7°C to recover this fungus in pure cultures. Thus, mycological investigations of WNS are still in their infancy. An alternative approach for confirming the diagnosis of WNS in bats was recently proposed by means of histopathological criteria [19]. The approach allows diagnosis to be established in symptomatic animals, and it should prove valuable in future surveys that seek to estimate the extent of disease among bat populations. Similar availability of genetic and possibly serological tools can be used for convenient and alternate confirmations of WNS in the affected bats. However, the recovery of the fungus in pure culture, and subsequent characterizations of the fungus, will be critical for achieving an understanding of WNS and bat mortality, and if we are to devise control measures.

Our group has engaged in mycological investigations, parallel to the studies of Blehert et al. [14]. Our findings provide strong evidence for an etiologic role of *G. destructans* in bat WNS. (i) Direct smears from bat snouts, Periodic Acid Schiff-stained tissue sections from infected tissues, and scanning electron micrographs of bat tissues all showed fungal structures similar to those of *G. destructans* (ii) *G. destructans* DNA was directly amplified from infected bat tissues (iii) Isolations of *G. destructans* in cultures from infected bat tissues showed 100% DNA match with the fungus present in positive tissue samples (iv) RAPD patterns for all *G. destructans* cultures isolated from two sites were indistinguishable (v) The fungal isolates showed psychrophilic growth (vi) We identified *in vitro* proteolytic activities suggestive of known fungal pathogenic traits in *G. destructans*. These findings should help underpin further study of the role of *G. destructans* in bat WNS and mass mortality.

## Results

### Microscopy of affected tissues

**Figure 1:**
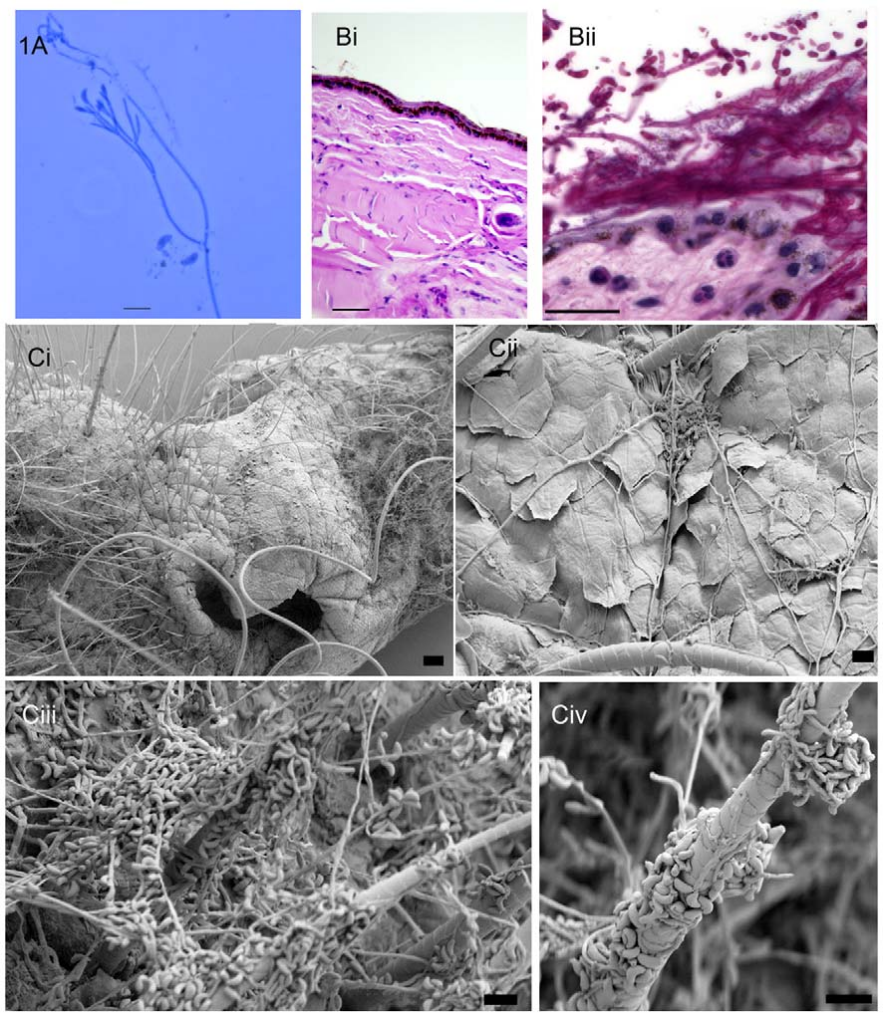
Microscopic and histopathological evidence of *G. destructans* in bats with WNS. (A) Direct lactophenol cotton blue mount prepared from skin scrape taken from the muzzle of a little brown bat from Graphite Mine on April 6, 2008 revealed fungal hyphae and curved conidia, bar 10 µm. (B) Control, [Bi] and infected muzzle tissue section [Bii] stained with PAS revealed epidermal colonization by fungal hyphae and spores; the sample was from a little brown bat from Williams Hotel Mine on March 27, 2008. Notably, a few neutrophils are present in the underlying dermis (arrows), bar 10 µm. Bacteria are also seen in this sample (C). SEM photomicrograph of muzzle sample from bat from Williams Hotel Mine showing characteristic curved conidia and septate hyphae spread over bat skin tissues. Note heavy fungal growth with profuse curved conidia covering the skin and hair shaft (Ci, muzzle, bar 100 µm; Cii, higher magnification of a portion of muzzle, bar 10 µm; Ciii & Cvi, higher magnifications, bar 10 µm).

Skin scrapes were taken from the muzzles of four little brown bats from the Graphite Mine on April 4, 2008. Lactophenol cotton blue mounts of these specimens showed curved conidia characteristic of ascomycetes fungi (Fig. 1A). Sections of skin from muzzles of four humanely euthanized little brown bats collected from the Williams Hotel Mine on March 27, 2008, showed epidermal colonization with focal dermatitis including a few neutrophils in the underlying dermis and fungal hyphae and spores intermixed with bacteria near the surface (Fig. 1Bi, ii). Pathology and virology studies carried out in parallel did not reveal any known bacterial, viral, or parasitic pathogens (details not shown). The evidence for the presence of a mycelial fungus in affected areas of the infected bats was obtained when tissue samples from the Williams Hotel Mine were examined by SEM; this imaging method revealed abundant fungal growth on skin and hair shafts (Fig. 1Ci-iv). The above observations led us to focus on the recovery of the mold from bat tissues and environmental samples.

### Fungal isolations

**Figure 2:**
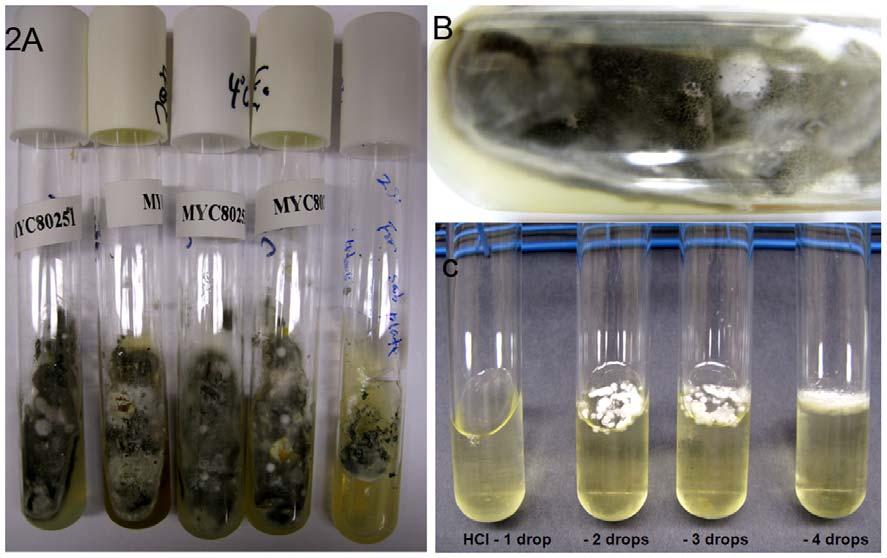
*G. destructans* in culture from bat tissues. (A). Original culture tubes of Sabouraud agar supplemented with nine antibiotics and incubated at 4°C for six- or eight-weeks; notice the profuse growth of *G. destructans* strains. (B) Some fungal contamination on individual isolates was visible as depicted in the close-up of a culture tube. (C) Enrichment and recovery of pure fungal colonies by treating a culture contaminated with bacteria with hydrochloric acid.

Initially, all submitted specimens were processed according to the protocols routinely followed by the Mycology Laboratory of the Wadsworth Center, for the recovery of human pathogenic fungi. Thus, Sabouraud agar with antibiotics and Mycosel agar were used for isolation of fungi at 30°C. All of the cultures were either quickly overgrown with bacterial contaminants or yielded common saprobic fungi that did not match the spore pattern seen in the initial microscopic analysis of bat specimens. We obtained similar results when environmental samples were processed on the above media or on Rose Bengal agar. Thereafter, we decided to fortify Sabouraud agar with multiple antibacterials, to discontinue the use of cycloheximide, and to incubate all inoculated tubes at 4°C, in an effort to simulate conditions in caves. After 4 weeks, a few tubes yielded evidence of a slow-growing fungus; the fungus covered the entire tube by 8 weeks (Fig. 2A). Six inoculated tubes yielded identical isolates. However, these tubes still carried a few contaminant fungi and bacteria (Fig. 2B). Fungal isolates were purified by dilution plating of fungal growth from initial culture tubes so as to obtain pure colonies; bacterial contaminants were removed by hydrochloric acid treatment per a procedure customarily followed in the Mycology Laboratory of the Wadsworth Center (Fig. 2C). Finally, we were able to purify five of the six isolates of the fungus from bat tissues; the sixth isolate was lost due to contamination of stock cultures.

### Fungal identification

**Figure 3:**
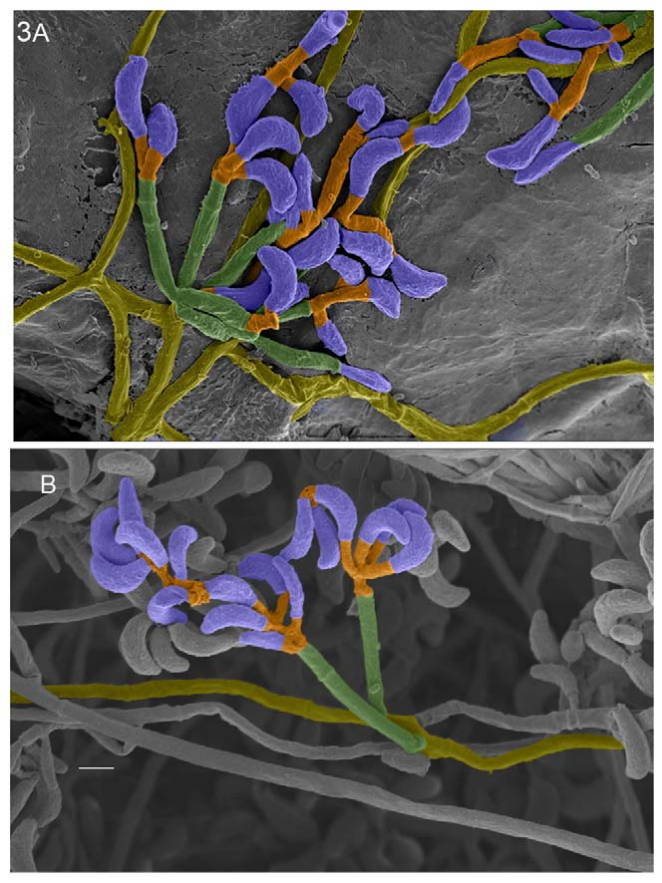
*G. destructans* in bat tissues and culture are similar. (A) SEM of photomicrograph prepared from bat tissues samples, examined from Fig. 1C at high magnification, showed fungal hyphae and spores on the surface. (B) SEM photomicrograph prepared from *G. destructans* culture isolated from bat tissue samples collected from Williams Hotel Mine; note curved conidia borne in whorls on septate hyphae; this pattern is similar to SEM image in Fig. 3A, bar is 2 µm. All images are pseudo-colored in Adobe Photoshop 9.0.

Initial observations of purified fungal colonies from bat tissues, on Sabouraud dextrose agar and potato dextrose agar, showed septate hyphae with abundant conidia borne directly on hyphae without any fruiting bodies (Fig. 3B). We compared the colony characteristics and spore formation against standard identification keys, and concluded that the isolates most closely resembled hyphomycete fungus classified as *Geomyces* sp. [16], [20]. Further evidence for involvement of *Geomyces* sp. in WNS was obtained frin SEM: hyphae and spores from the pure cultures showed exact matches with the fungal hyphae and spores imaged by SEM in tissues from an infected bat (Fig. 3A). Interestingly, one member of the genus, *G. pannorum*, was already suggested to be involved in some humans and animal mycoses [21], [22], [23], [24]. However, the curved conidia seen in bat isolates were distinct from the club-shaped conidia of *G. pannorum*, and no arthrospores were seen [20]. All of our initial identifications were tentatively termed *Geomyces* sp. More recently, the fungus has been delineated as a new species *G. destructans* [15]. Because the features in our isolates were identical to those of type specimen, we re-named all of our strains as *G. destructans*. We have deposited a representative strain (*G. destructans* MYC80251) in the CBS Fungal Collection, Utrecht, The Netherlands.

### DNA amplification, nucleotide sequencing and phylogenetic analyses

**Figure 4:**
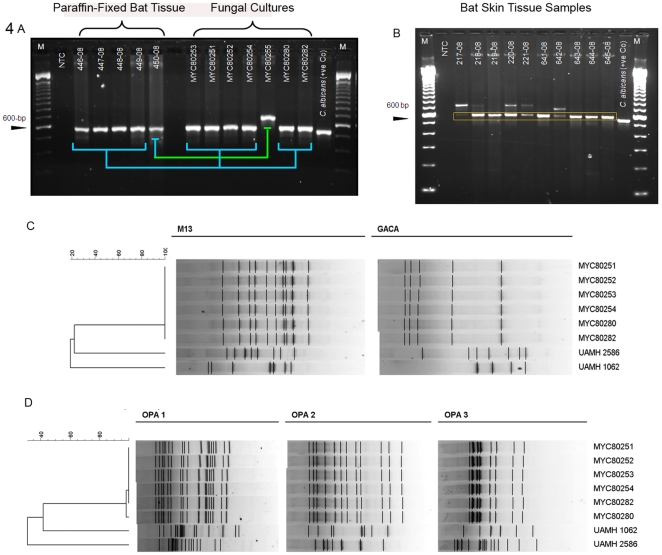
Molecular analysis of bat tissues and fungal cultures. (A) ITS PCR analysis of bat tissues and fungal cultures from DNA extracted from bat tissues and from pure *G. destructans* isolates. PCR amplification was carried out with primer set V47/V50. PCR amplicons were electrophoresed on 2% agarose gel, stained with ethidium bromide and photographed with a imaging software. Four bat tissues and respective fungal isolates showed perfect matches (blue connectors); one tissue DNA amplicon did not match with *G. destructans* amplicon obtained from pure culture (green connector). Also shown are amplicons from two additional *G. destructans* isolates (MYC80280, MYC80282) where corresponding tissues samples were not processed. (B) ITS PCR analysis of bat tissue samples positive for *G. destructans*. Ten bat tissues including five untreated samples and five paraffin-fixed samples were positive for *G. destructans* DNA (details in Table 1). (C-D) Molecular typing of *G. destructans* was performed with RAPD primers. (C) Results shown were obtained by PCR of fungal genomic DNA with M-13 and (GACA)_4_ primers, amplicons were run on 2% agarose gels and band patterns were used to construct dendrograms with Applied Math software. *Geomyces pannorum* (UAMH 1062 and UAMH 2586) were used as outgroup. (D) Results shown were obtained by PCR of genomic DNA with Operon Technology 10-mer primers OPA1, OPA2 and OPA3; outgroup strains are similar to panel in C. Genotyping with five different primers showed that all six *G. destructans* culture isolates obtained from two sites, approximately 200-km apart, had indistinguishable band patterns. These preliminary results raised the possibility of involvement of a single strain of *G. destructans* in the outbreak of WNS in bats in upstate NY.

Of 17 bat skin tissue samples available for DNA extraction and ITS PCR, 15 proved to be positive for fungal DNA (Table 1). When the PCR products were sequenced and BLAST-searched against GenBank, the closest identity (98%) was seen with *G. pannorum* strains. Therefore, we initially deposited our sequences in the database as *G. pannorum* (GenBank EU877917 –EU877931). For four bat samples, identical DNA band patterns were obtained for ITS-PCR analysis from matching tissues and pure cultures (Fig. 4A). Additional information, in Table 1 and Fig. 4B, indicates that 10 bat tissue samples comprising five untreated samples plus five paraffin-embedded samples were positive for *G. destructans* DNA. No corresponding culture isolations of *G. destructans* could be made from five untreated tissue samples, because the initial incubations in our laboratory had been at 30°C. Table 1 and Fig. 4B also show that five of 10 bat tissue samples yielded an amplicon beside the expected band for *G. destructans*. Each of these bands was approximately 650–700 bp in size. Sequencing and BLAST searching revealed that four of these amplicons matched a sequence from *Helicostylum elegans*, and the fifth amplicon matched a GenBank sequence deposited as *Mortierella* species. The significance of these additional bands is not clear at present.

**Table 1:** *G. destructans* work done at the Mycology Laboratory of the Wadsworth Center.

Collection Date	Location	Tissue Samples	Pathology#	Pathology Results	Mycology#	Incubation	Culture Results	ITS-PCR Tissue	ITS-PCR Culture	GenBank Acc. No.
2/26/08	WmsLake	Fixed &fresh	217-08	Lesional∫	MYC80127	30°C	–	+ +§	NA	GU944940
2/26/08	WmsLake	Fixed &fresh	218-08	Lesional∫	MYC80129	30°C	–	+ +§	NA	GU944941
2/26/08	WmsLake	Fixed &fresh	219-08	Lesional∫	MYC80131	30°C	–	+	NA	EU877928
2/26/08	WmsLake	Fixed &fresh	220-08	Lesional∫	MYC80133	30°C	–	+ +§	NA	GU944942
2/26/08	WmsLake	Fixed &fresh	221-08	Lesional∫	MYC80135	30°C	–	+ +§	NA	GU944943
2/26/08	WmsLake	Fixed only	641-08	Lesional∫	NA	NA	NA	+	NA	EU877929
2/26/08	WmsLake	Fixed only	642-08	Lesional∫	NA	NA	NA	+ +§	NA	GU944944
2/26/08	WmsLake	Fixed only	643-08	Lesional∫	NA	NA	NA	+	NA	EU877930
2/26/08	WmsLake	Fixed only	644-08	Lesional∫	NA	NA	NA	+	NA	EU877931
2/26/08	WmsLake	Fixed only	645-08	Lesional∫	NA	NA	NA	+	NA	GU944945
3/27/08	WmsHotel	Fixed &fresh	446-08	Lesional∫	MYC80251	4°C	**+**	+	+	EU877917, EU877923
3/27/08	WmsHotel	Fixed &fresh	447-08	Lesional∫	MYC80252	4°C	**+**	+	+	EU877918, EU877924
3/27/08	WmsHotel	Fixed &fresh	448-08	Lesional∫	MYC80253	4°C	**+**	+	+	EU877919, EU877925
3/27/08	WmsHotel	Fixed &fresh	449-08	Lesional∫	MYC80254	4°C	**+**	+	+	EU877920, EU877926
3/27/08	WmsHotel	Fixed &fresh	450-08	Lesional∫	MYC80255	4°C	–	+	–	EU877927
4/8/08	Graphite	Swab	–	NA	MYC80280	4°C	**+**	ND	+	EU877921
4/8/08	Graphite	Swab	–	NA	MYC80282	4°C	**+**	ND	+	EU877922

The data include results of PCR on bat tissue samples collected from little brown bats (*Myotis lucifugus*), culture information obtained with standard protocols for the recovery of human pathogenic fungi and a modified culture procedure to recover this psychrophilic fungus at 4°C.

Wms Lake, Williams Lake Mine; Wms Hotel, Williams Hotel Mine; NA, Not applicable (paraffin-fixed tissue); ND, Not done

∫ Gross and microscopic fungal infection in skin of muzzle and wing membranes, except 641- wing membrane only

§ Tissue DNA produced two amplicons with one positive for *G. destructans* and second positive for *Helicostylum elegans* (217-08, 218-08, 220-08, 221-08) and *Mortierella* sp. (642-08).

**Figure 5:**
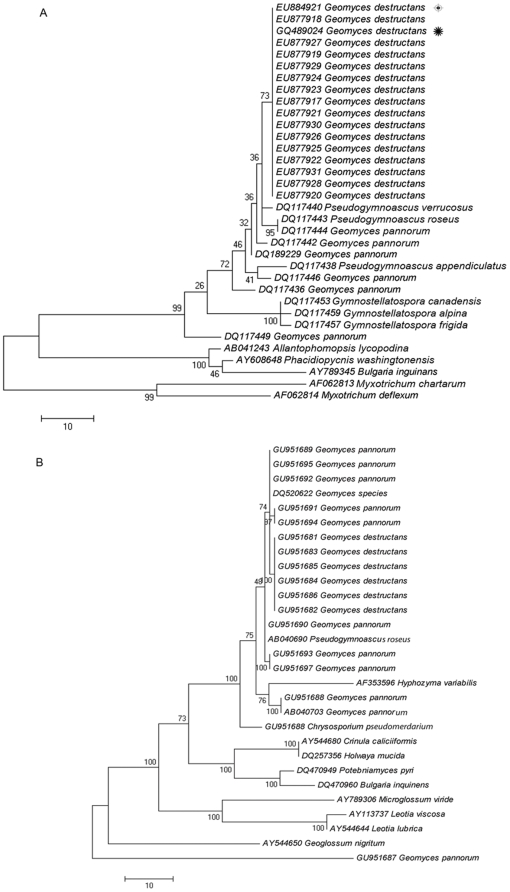
Phylogenetic analysis of nucleotide sequences from *G. destructans*. (A) Phylogenetic tree was constructed by parsimony analysis of ITS sequences. The evolutionary history of representative isolates of *G. destructans* from this study and the sequences in the databases were inferred using the Maximum Parsimony method and bootstrap consensus tree from 1000 replicates conducted in MEGA 4.1 [54]. After elimination of gaps and missing data, the dataset contained 448 positions of which 109 were parsimony informative. Percentage of replicate trees shown indicate clustering of associated tax in 1000 bootstrap replicates. Asterisks denote sequences deposited by other investigators from bats with WNS in US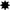 [14] and France 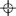 [18]. (B) Phylogenetic tree constructed by parsimony analysis of 28S ribosomal sequences. The evolutionary history of representative isolates of *G. destructans* from this study and additional related fungi sequenced in our laboratory, were inferred using the Maximum Parsimony method and bootstrap consensus tree from 1000 replicates conducted in MEGA 4.1. After elimination of gaps and missing data, the dataset contained 537 positions of which 88 were parsimony informative. The consensus phylogenetic tree shown was inferred from 94 most parsimonious trees.

Initial phylogenetic analyses of fungal ITS sequences from bat- derived pure cultures and from tissue samples supported close relationship with *G. pannorum* sequences in GenBank, but the sequences from these samples formed a distinct clade with high bootstrap values (Fig. 5A). Our sequences from bat isolates and from bat tissue samples also showed perfect matches with sequences deposited earlier by Blehert et al. [14] and with more recent sequences from France, deposited by Puechmaille et al. [18]. All of our isolates are now identified in GenBank as *G. destructans*. Additional evidence for the taxonomic grouping is provided by phylogenetic analyses, based on 28S ribosomal RNA gene sequences that again yielded distinct but related clades of *G. pannorum* and *G. destructans* (Fig. 5B).

### Molecular typing by RAPD

RAPD molecular typing was used to assess whether single or multiple strains of *G. destructans* were involved in the local occurrence of bat WNS. Genotyping with five different primers showed that all six *G. destructans* culture isolates obtained from two sites, approximately 200-km apart, had indistinguishable band patterns. These band patterns were distinct from two *G. pannorum* isolates used as an outgroup in this analysis (Fig. 4C). Our preliminary results raised the possibility of involvement of a single strain of *G. destructans* in the outbreak of WNS in bats in upstate NY.

### *G. destructans* characterization

**Figure 6:**
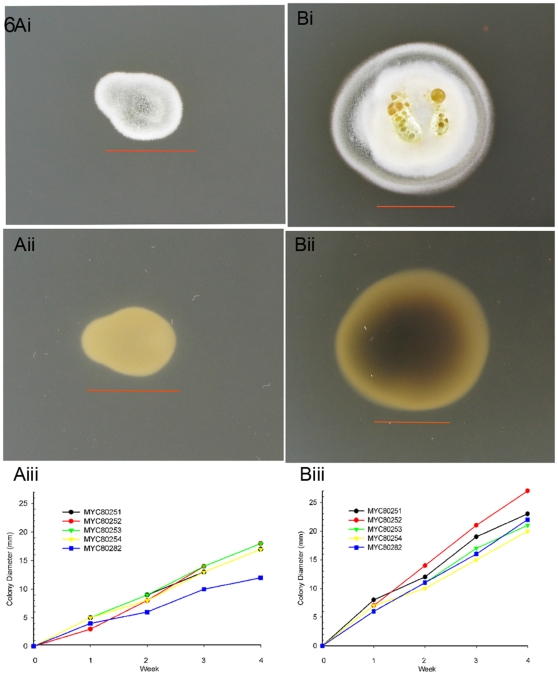
Growth characteristics of *G. destructans* isolates. Colony morphology and growth rates were compared on Sabouraud dextrose agar and potato dextrose agar at -10°C, 4°C, 15°C, and 25°C. (A). Close up of fungal colonies of the isolate MYC80254 incubated at 4°C (Fig. 6A) and 15°C (Fig. 6B) for 28 days on potato dextrose agar, marker 10 mm. The initial colony appearance was white, velvety, glabrous turning grayish green, powdery in texture. Reverse with no pigmentation initially (Fig. 6Ai) later on revealing diffusible dark brown pigment (Fig. 6Bii). Older colony also exhibited exudates on surface, marker 10 mm. (Fig. 6Bi). Colony diameters of five *G. destructans* strains isolated from bat tissues and incubated for 28 days on Sabouraud dextrose agar at 4°C and 15°C. Exponential growth was seen at both temperatures with larger colony diameters at 15°C (Fig. 6Biii) than at 4°C (Fig. 6Aiii). The results represent average of two separate experiments. There was no growth in cultures incubated concurrently at −10°C or at 25°C (data not shown).

**Figure 7:**
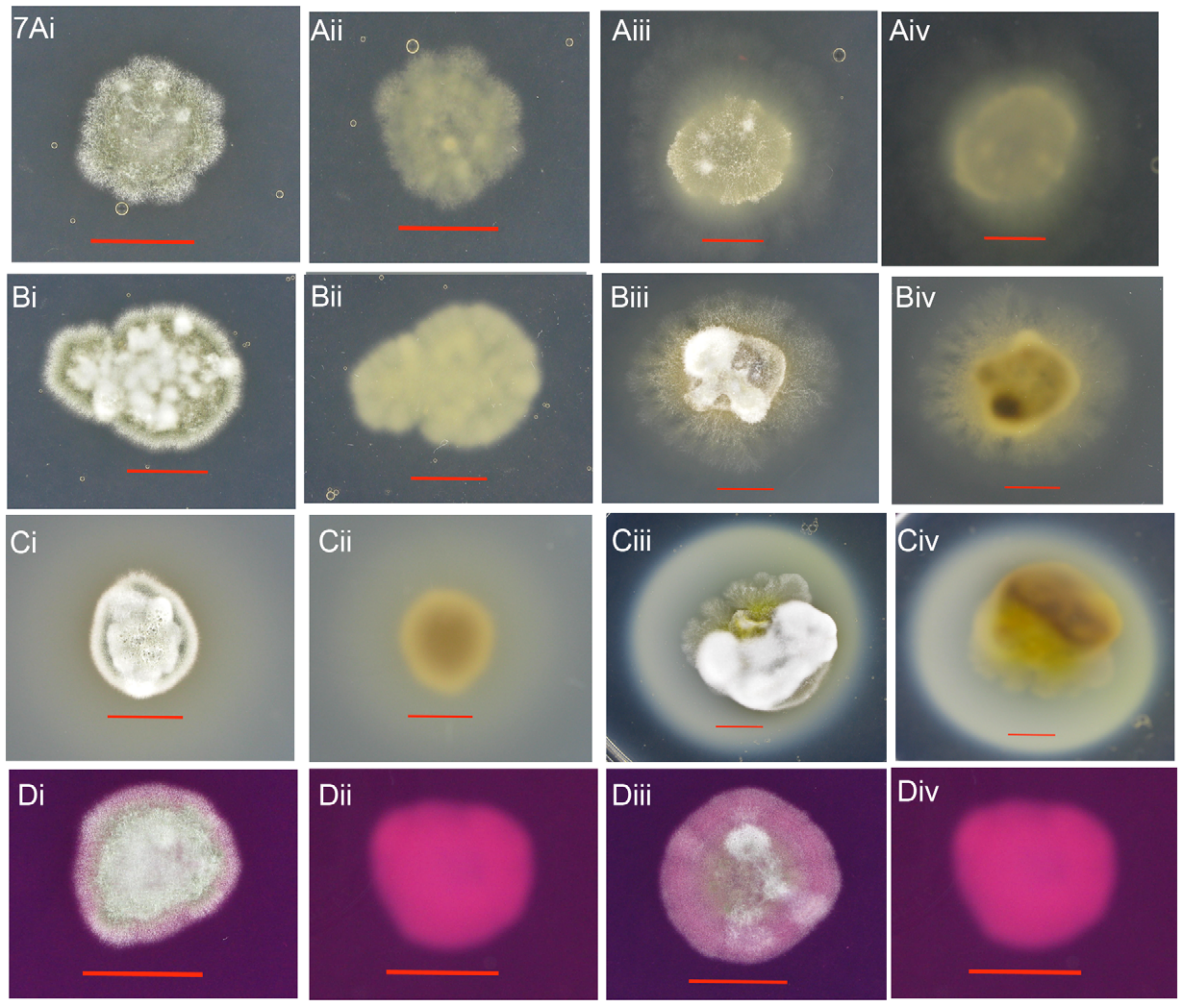
*G. destructans* proteolytic activities. Results from a representative strain, *G. destructans* MYC80-0251, showed secretory proteases after 28-days growth on albumin agar (Ai-ii, 4°C front & reverse; Aiii-iv, 15°C front & reverse), Casein agar(Bi-ii, 4°C front & reverse; Biii-iv, 15°C front & reverse), Geleatin agar(Ci-ii, 4°C front & reverse; Ciii-iv, 15°C front & reverse) or Urea agar at 4°C and 15° (Di-ii, 4°C front & reverse; Diii-iv, 15°C front & reverse), marker is 10 mm. Similar patterns of secretory proteases were seen with remaining four *G. destructans* strains.

The psychrophilic nature of the recovered fungal strains was tested at -10°C, 4°C, 15°C and 25°C by incubation of point-inoculated colonies on Sabouraud dextrose agar and potato dextrose agar. Good growth was seen both at 4°C and 15°C (Fig. 6) while no growth was visible either at -10°C or 25°C (data not shown). A comparison of growth rates, between 4°C (Fig. 6Aiii) and 15°C (Fig. 6Biii), revealed that the latter was optimal for fungal growth. The ability of the fungus to produce proteolytic and hydrolyzing enzymes was tested by API-ZYM tests. *Geomyces destructans* isolates produced acid phosphatase, alkaline phosphatase, N-acetyl- β-glucosaminidase, β- glucosidase, esterase, esterase lipase, lipase, leucine arylamidase, naphthol-AS-B1-phophohydrolase, and vailine arylamidase. The fungus did not produce cystine arylamidase, trypsin, α-chymotrypsin, α-galactosidase, β-galactosidase, β-glucoronidase, α-glucosidase, β-glucosidase, α-mannosidase, and α-fucosidase. Plate assays showed that *G. destructans* secreted proteinases when albumin, casein or gelatin was used as substrates; the fungus was also positive for urease on urea agar (Fig. 7). Surprisingly, no growth was seen on egg yolk agar, which was used to test for phospholipase activity (data not shown).

## Discussion

The current study has provided a set of complementary observations that strengthen the evidence for an etiologic role of *G. destructans* in bat WNS. (i) Direct smears from bat snouts, Periodic Acid Schiff-stained tissue sections from infected tissues, and scanning electron micrographs of bat tissues all showed fungal structures similar to those of *G. destructans* (ii) *G. destructans* DNA was directly amplified from infected bat tissues (iii) Isolations of *G. destructans* in cultures from infected bat tissues showed 100% DNA match with the fungus present in positive tissue samples (iv) RAPD patterns for all *G. destructans* cultures isolated from two sites were indistinguishable (v) The fungal isolates showed psychrophilic growth (vi) We identified *in vitro* proteolytic activities suggestive of known fungal pathogenic traits in *G. destructans*. Our findings that *G. destructans* in bat tissues can be verified by DNA analysis and culture from the same sample, suggest that PCR- nucleotide sequencing can suffice as a screening test for confirming the presence of fungus in bats with WNS.

It is striking that the successful isolations of *G. destructans* were very few in number, relative to the large number of bat samples examined in this study. Similarly, no fungal isolations could be made from many environmental samples that had been collected from various sites. The low recovery is consistent with the isolations rates documented in previous brief reports on *G. destructans* from bats both the USA and France [14], [18]. It is possible that the initial processingof skin and environmental samples at 30°C, a temperature that is optimal for recovery of human and animal pathogenic fungi, proved deleterious to any *G. destructans* that was present in these samples. Additionally, the use of routine culture media appeared inadequate for G. *destructans* recovery, and so that additional measures were needed before colonies could be purified. It is additionally possible that the particular soil and debris samples analyzed were not optimal for the isolation of *G. destructans*; many human and animal pathogenic fungi have narrow, specific environmental niches, such that and their natural isolations still remain a rare event despite the development of selective media [25], [26], [27], [28]. Use of selective isolation media incubated under psychrophilic condition, or additional processing of samples prior to inoculation, could be necessary if we are to consistently recover *G. destructans* from the infected animals and their surroundings. Our present findings provide clues as to the modifications that could facilitate future investigations.

Our data on genotyping suggest the existence of a clonal *G. destructans* population in our sampled areas over a 200-km distance. The next logical step would be to find out whether *G. destructans* isolates were native to the hibernacula, or instead a recent ‘hitch-hiker’ into new environment, as is known to be the case for some fungi in Antarctica [29]. Such a study would be important, to account for why WNS suddenly appeared in hibernacula that had no prior history of it across three decades of recorded observations. Some strains of fungi found in the Antarctic strains have been shown to have clonal populations, an attribute not seen in strains collected from other environments such alpine, temperate or tropical areas [30], [31]. We are currently engaged in studies of fungal population genetics, to delineate the likely origin of our *G. destructans* isolates. The results from these studies should aid in the evaluation of potential control measures, and in the selection of ‘host strain’ for detailed laboratory experiments.

A wide variety of psychrophilic fungal species have been reported from surveys carried out in Arctic and Antarctic environments, and permafrost zones in Russia [29], [32], [33], [34]. Additional lineages of psychrophilic fungi certainly exist as some of them are distributed more widely among temperate climates [35], [36]. No known primary human or animal pathogenic fungi number among the known psychrophiles. However, a number of *Chrysosporium* species and *Geomyces* species recovered from extreme low temperatures are known to be capable of degrading hair, skin, and nails [37]. Similarly, a few reports describe *Geomyces* species, especially *G. pannorum*, as causal agents of human and animal infections [20], [21], [22], [23], [24], [38]. As yet, no human ailments have been reported from areas with WNS and bat mortality. We do not know whether the psychrophilic or the psychrotolerant fungi are pathogenic, or whether their adaptation to the cold is important in the maintenance or enhancement of any pathogenic attributes. In view of our preliminary data, *G. destructans* proteolytic attributes could play a role in pathogenesis of WNS. It is relevant to recall that grooming, including the allogrooming and nose rubbing common among bats, could enhance traumatic entry of the fungus into affected tissues of bats within a given colony [39]. In such a scenario, the *G. destructans* proteolytic enzymes identified in the present study could enhance fungal growth and dissemination. Importantly, many of the secretory proteases identified in *G. destructans* are implicated as virulence traits in a wide variety of microbial pathogens [40]. The skin lesions of WNS-affected bats that were observed by us and others differed from dermatophytosis (ringworm) in animals; the latter condition presents with few to scant fungal elements internal or external to hair shafts, and pronounced inflammation, most commonly granulomatous folliculitis [19], [41]. In contrast, the bats in the current outbreak showed numerous fungal hyphae and spores in their skin, but with scant inflammation. Possibly, such lack of pronounced inflammation associated with fungal invasion observed in this study could be due to differences in general immune responses in bats relative to other mammals or more likely due to a dormant immune system in hibernating animals, which prevents mounting of such a response [42], [43], [44].

Currently, WNS is thought to cause a major disturbance of deep torpor or hibernation; the affected animals are seen to repeatedly groomed areas around their nostrils [14], [18], [19]. Active grooming is a normal behavior for ectoparasite removal, but not during hibernation [39], [45]. It is possible that abnormal grooming could affect the animal's general fitness by depleting energy reserves, and this could decrease long-term survival [46]. Another contributing factor to decreased survival could be that disturbance of the normal hibernation pattern leads to pre-mature departure of the affected animals from the hibernacula. Since insects would not yet have appeared in the surrounding areas, there was no quick replenishment of nutrients and many animals would starve. However, this scenario appears insufficient to account for the great number of dead animals; many carcasses are found deep inside the affected caves and caverns. Therefore, the results obtained thus far, by us and others, do not provide a direct explanation for the observed mass mortality of bats nor do they establish a direct causal link between WNS and bat deaths. It is also not known if the fungus causing WNS produces a toxin or other metabolites that could cause systemic organ failures or malfunctions in the infected bats. Not all bats examined from the affected hibernacula show all signs of WNS, thus raising the possibility that there exists a subclinical form or stage of WNS, which may or may not have originated in the hibernacula. Moreover, the carriage of *G. destructans* by a healthy *M. myotis* without any discernible deleterious effects, as documented in the recent report from France raises issues related to host immunity, fungal and other microbial flora, and the role of environmental factors [18]. Further investigations are warranted to establish whether WNS is a symptom of or a trigger for mass mortality in bats.

## Materials and Methods

### Bat specimens

Seventy-four bat specimens were processed for histology and mycology studies including 55 little brown bats (*Myotis lucifugus*), two northern long-eared bats (*Myotis septentrionalis*); two small-footed bats (*Myotis leibii*), and one Eastern Pipistrelle (*Perimyotis subflavus*). Twenty-three of 47 bats whose sex was recorded were females, and 24 were males. The collection sites were Hailes Cave, Williams Hotel Mine, Williams Preserve Mine, Williams Lake Mine, Clarksville Cave, Bitting House, Martins Mine, and Graphite Mine within a 200-km radius from Albany, NY (Fig. S1). Twenty-six environmental samples, comprising mainly soil and debris, were collected from some of these sites and also processed. Lactophenol- cotton blue wet mounts were made from gross skin lesions of the muzzles of four bats from Graphite Mine. DNA from 17 of these bat skin tissue samples were processed for fungal identification by PCR –nucleotide sequencing. All bats were tested for rabies by fluorescent antibody (FA) staining of brain smears at the Rabies Laboratory of the Wadsworth Center.

### Fungal isolation and characterization

Sabouraud dextrose agar (SDA), Mycosel ® agar, SDA fortified with an enhanced panel of antibacterials (amikacin, ampicillin, carbenicillin, cephelixin, cefazolin, colistin methanesulfonate, kanamycin, ofloxacin, streptomycin, tetracycline, and vancomycin), and Rose Bengal agar were used for the isolation and purification of fungi from the bat tissues and environmental specimens. Potato dextrose agar (PDA) was used to induce spore formation. Standard mycology techniques were used for the study of fungal growth on culture media, temperature requirements, and microscopic studies [20], [47], [48]. Proteolytic enzyme activities were tested with an API ZYM test kit (bioMerieux SA, 69280 Marcy-l'Etoile, France); another medium used was based upon 2% agar containing either 2% (w/v) bovine serum albumin, casein or gelatin [49], [50]. Phospholipase activity was measured by a plate method using 8% sterile egg yolk [51]. Light microscopic and scanning electron microscopy (SEM) analyses were carried out per protocols routinely used in our laboratory [52].

### DNA Isolation, PCR, sequencing and analysis

Genomic DNA from various isolates of *Geomyces destructans,* and from *Geomyces pannorum* (University of Alberta Mold Herbarium, Edmonton, Alberta, Canada), were isolated in a BSL-2 cabinet. In brief, fungal growth (approximately 2–5 mm) was suspended in 0.5 ml of DNA extraction buffer (10 mM Tris-HCl Ph 7.5; 1% SDS; 100 mM EDTA; 2% TritoN X 100, and 100 mM NaCl) containing 2 g of acid-washed glass beads. The fungal-glass bead suspension was incubated at 70°C for 1 h and then disrupted in a cell disrupter (Vortex-Genie 2; Fisher Scientific, Pittsburgh, PA) for 20 min. DNA was extracted by a conventional phenol-chloroform procedure, followed by ethanol precipitation. The DNA was purified with 70% ethanol, air-dried, and re-suspended in 50 µl of sterilized MilliQ water containing 200 µg/ml RNase. DNA was finally passed through a PERFORMA® spin column (Edge BioSystems, Gaithersburg, MD), to remove any trace amount of solvents, fungal pigments, and low molecular weight reagents encountered during DNA precipitation steps. DNA from bat skin tissue samples (untreated and paraffin fixed) was extracted with the QIAamp DNA FFPE kit (Quiagen, Valencia, CA) per manufacturer's instructions with minor modifications. After the tissue lysis step, glass beads were added, and the mixture was disrupted in a cell disrupter for 20 min. The internal transcribed spacer (ITS) regions (ITS1, 5.8S, and ITS2) and D1/D2 region of the gene encoding 28S RNA of the large subunit (LSU) were amplified using the respective primer sets V47 (ITS1) 5′- TCCGTAGGTGAACCTGCGG – 3′, and V50 (ITS4) 5′ – TCCTCCGCTTATTGATATGC – 3′ and primer set V1798 5′ – GCATATCAATAAGCGGAGGAAAAG-3′, and V1799 5′ – GGTCCGTGTTTCAAGACGG -3′ [53]. PCR was performed in 25 µl of a reaction mixture containing 2 µl of DNA (10–50 ng/µl), 1× buffer containing 1.5 mM MgCl_2_, 0.02% bovine serum albumin (BSA), 0.2 mM of each dNTP, 0.4 µM of each primer, and 0.5 U JumpStart™ KlenTaq® LA DNA polymerase (SigmaAldrich, St. Louis, MO). PCR set up included initial denaturation at 94°C for 1 min, followed by 30 cycles of denaturation at 94°C for 1 min, annealing at 55°C for 1 min, and extension at 68°C for 1 min and a final extension step of 68°C for 3 min. The PCR products were electrophoresed on 2% agarose in Tris-borate-EDTA buffer, pH 8.3, and amplification products were stained with ethidium bromide and photographed with a visible-UV imaging system (Bio-Rad, Hercules, CA). Amplicons were purified with ExoSAP-IT (USB Corp., Cleveland, OH) and subjected to nucleotide sequencing of both strands, with the primers listed above. The Wadsworth Center Molecular Genetics Core facility performed dideoxy sequencing, using an ABI BigDye Terminator version 3.1 cycle sequencing kit with an ABI 3130 or 3730 DNA analyzer (Applied Biosystems, Foster City, CA). The nucleotide sequences were assembled and edited for accuracy with the Sequencher Software 4.6 (Gene Codes Corp., Ann Arbor, MI). Phylogenetic and molecular evolutionary analyses were conducted with the MEGA version 4.1 [54].

### Molecular typing by random amplification of polymorphic DNA (RAPD)

Molecular typing was done with five random probes: a 15-bp minisatellite probe from M13 bacteriophage (5′ GAGGGTGGCGGTTCT 3′); a microsatellite repeat (GACA)_4_ probe; and three 10-mer probes namely, OPA 1 (5′-AATCGGGCTG-3′), OPA 2 (5′-GTGATCGCAG-3′) and OPA 3 (5′-GACCGCTTGT), from Eurofins MWG Operon (Huntsville, AL) [55]. PCR was performed in a 20-µl reaction volume containing 2 µl of DNA (50 ng/µl), 1× PCR buffer, 2.5 mM MgCl_2_, 0.02% BSA, 0.25 mM of each dNTP, 2.5 µl of each primer, and 0.4 U of AmpliTaq DNA polymerase (Applied Biosystems). Single primers were used in the PCR, according to the protocol of Meyer et al.[56]. The PCR steps were initial denaturation at 94°C for 5 min, followed by 35 cycles of denaturation at 94°C for 1 min, annealing at 50°C for 1 min, extension at 72°C for 2 min, and final extension step of 72°C for 8 min. For each of the OPA primers, 45 cycles were used, with annealing at 38°C. PCR amplicons were separated on a 2% agarose gel in TBE buffer, stained with ethidium bromide and photographed with a visible-UV imaging system (Bio-Rad, Hercules, CA). The PCR amplicon patterns were analyzed with Bionumerics software (Applied Maths Inc., Austin, TX). Dendrograms were created by use of unweighted pair group similarity and arithmetic mean with Dice coefficients, and the position tolerance was set at 1.1% [57].

## Supporting Information

Figure S1 Current estimates of the origin and spread of WNS and bat mortality in the United States. Counties are categorized as “Confirmed” or “Likely” by each State agency. All states have confirmed the initial detection within their jurisdiction through submission of specimens validated by a laboratory. Continued confirmation of new counties within a state includes laboratory validation of specimens and/or confirmed by observed clinical field signs within a hibernaculum by state biologists. The “Likely” category is often used prior to receiving confirmation of submitted lab specimens or when unusual winter bat activity is observed on the landscape but observed clinical signs or laboratory specimens cannot be confirmed.
(4.55 MB DOC)
