# Supplementary material for: Morphological and Molecular Characterizations of Psychrophilic Fungus Geomyces destructans from New York Bats with White Nose Syndrome (WNS)
Source: PLoS One. 2010 May 24;5(5):e10783. doi: 10.1371/journal.pone.0010783 (PMC2875398; doi:10.1371/journal.pone.0010783)

**S1 Supporting Information**


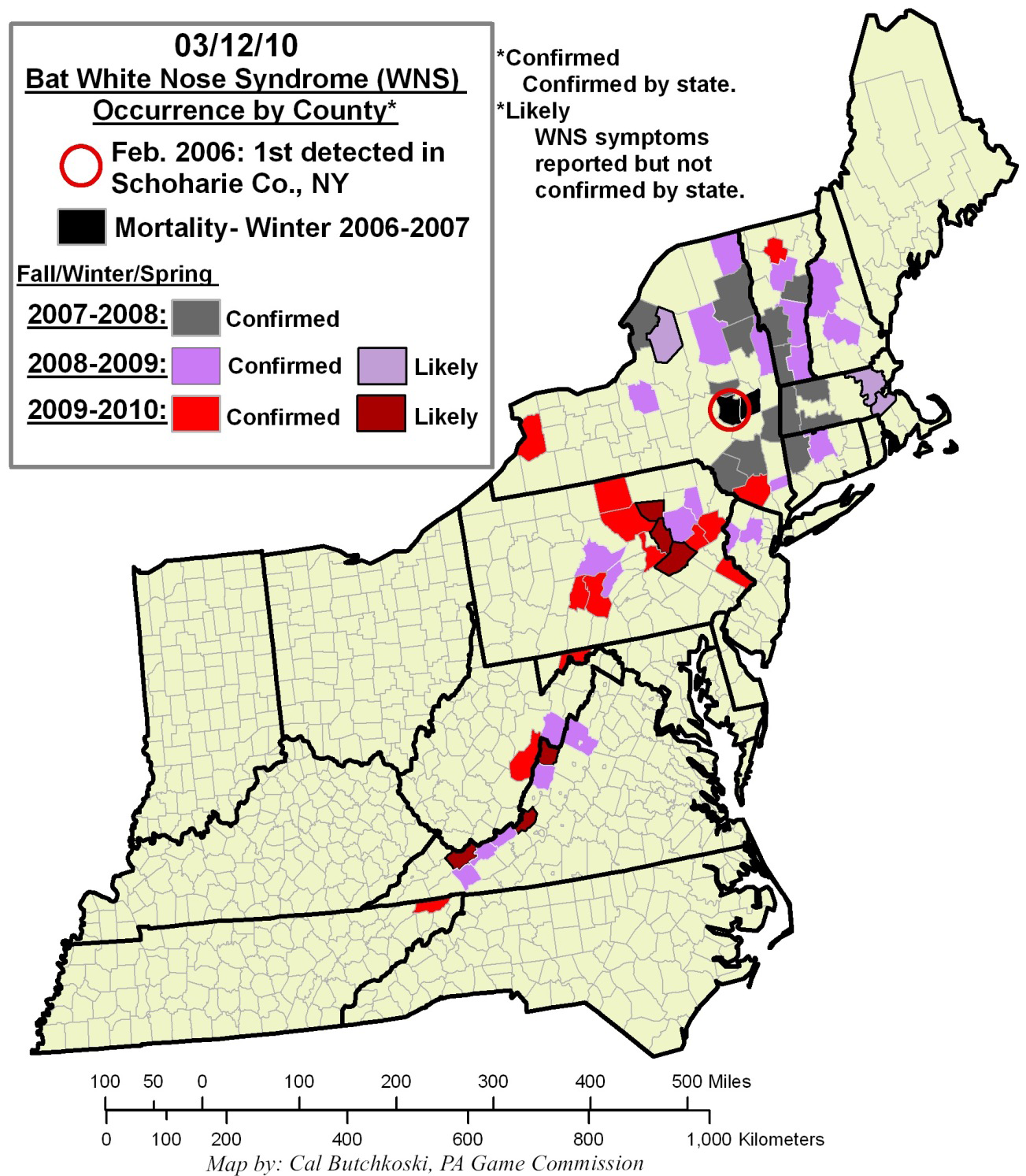
**Figure S1.** **Current estimates of the origin and spread of WNS and bat mortality in the United States.** Counties are categorized as “Confirmed” or “Likely” by each State agency. All states have confirmed the initial detection within their jurisdiction through submission of specimens validated by a laboratory. Continued confirmation of new counties within a state includes laboratory validation of specimens and/or confirmed by observed clinical field signs within a hibernaculum by state biologists. The “Likely” category is often used prior to receiving confirmation of submitted lab specimens or when unusual winter bat activity is observed on the landscape but observed clinical signs or laboratory specimens cannot be confirmed.

**Figure S2**. **Little brown bats (*Myotis lucifagus*) with White Nose Syndrome (WNS)**. The animals were photographed on March 20, 2008 from Graphite Mines, New York.


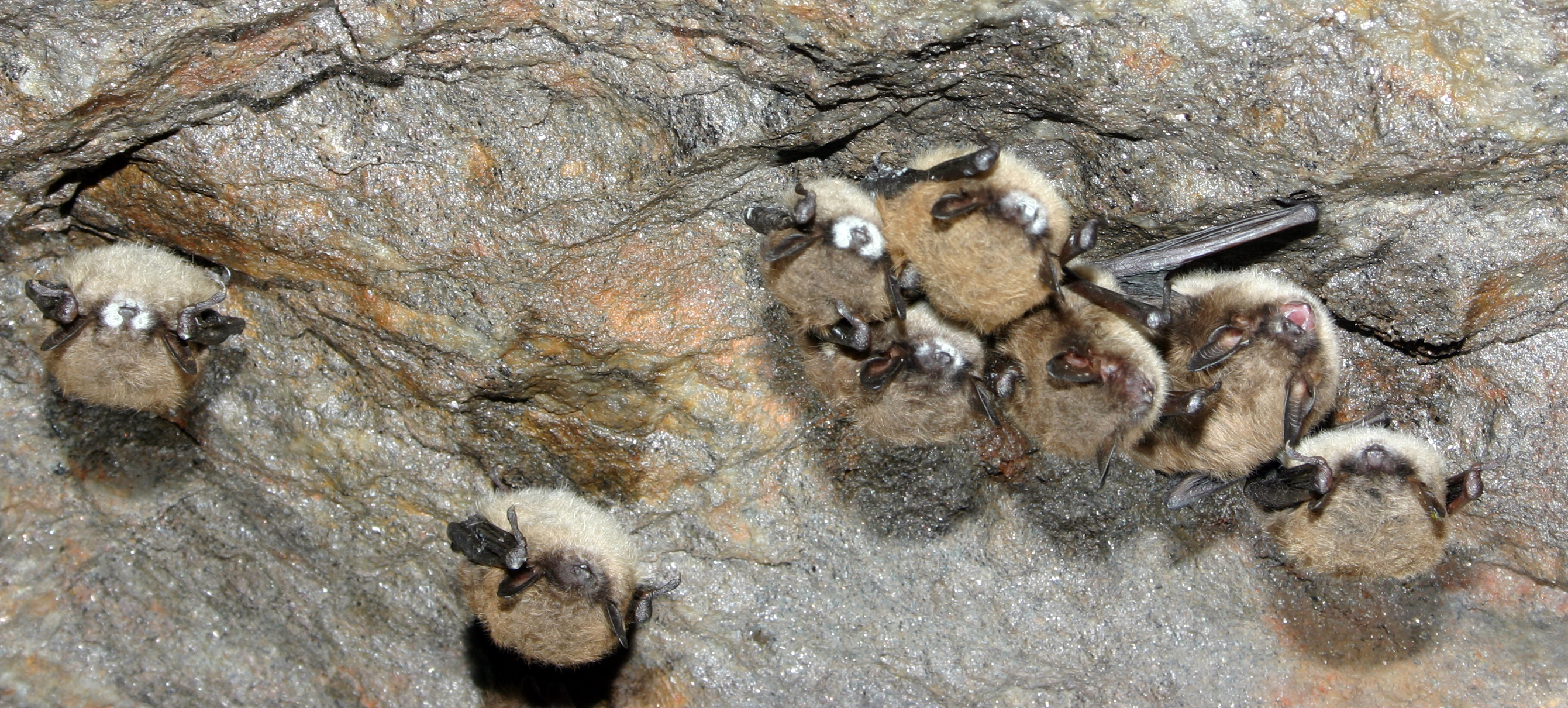

Supplement: Figure S1 — Current estimates of the origin and spread of WNS and bat mortality in the United States. Counties are categorized as “Confirmed” or “Likely” by each State agency. All states have confirmed the initial detection within their jurisdiction through submission of specimens validated by a laboratory. Continued confirmation of new counties within a state includes laboratory validation of specimens and/or confirmed by observed clinical field signs within a hibernaculum by state biologists. The “Likely” category is often used prior to receiving confirmation of submitted lab specimens or when unusual winter bat activity is observed on the landscape but observed clinical signs or laboratory specimens cannot be confirmed. (4.55 MB DOC) [file pone.0010783.s001.doc]
